# Beyond global North–led response: South–South collaboration at the frontline of Marburg virus outbreak control

**DOI:** 10.1371/journal.pgph.0006515

**Published:** 2026-06-03

**Authors:** Tsion Firew, Finot Debebe, Woldesenbet Waganew, Tesfaye Getachew Shawel, Lemlem Beza, Menelas Nkeshimana

**Affiliations:** 1 Africa Health Sciences University and King Faisal Hospital Rwanda, Kigali, Rwanda; 2 Ministry of Health of Rwanda, Kigali, Rwanda; 3 Ronald Perlman Department of Emergency Medicine, New York University, New York, New York, United States of America; 4 Addis Ababa University, College of Health Sciences, Addis Ababa, Ethiopia; 5 St. Paul’s Hospital Millennium Medical College, Addis Ababa, Ethiopia; 6 Department of Internal Medicine, University of Rwanda, College of Medicine and Health Sciences, Kigali, Rwanda; None, UNITED STATES OF AMERICA

## Introduction

One year after Rwandan authorities declared the end of their Marburg virus disease (MVD) outbreak, the Ethiopian Ministry of Health confirmed a new outbreak on November 14, 2025.

Since the 2024 outbreak in Rwanda, the East African region has faced successive Marburg outbreaks, including in Tanzania and subsequently Ethiopia, underscoring the persistent threat posed by filoviruses [1,2]. Over the past decade, similar outbreaks have occurred in Ghana, the Democratic Republic of the Congo, Kenya, Uganda, and Equatorial Guinea [1,2].

Marburg virus disease, caused by a filovirus closely related to Ebola, has historically been associated with case fatality rates (CFR) approaching 90%. The combination of high mortality and a lack of approved therapeutics renders MVD one of the deadliest viral threats to humanity. This risk is particularly acute in low-income countries, where resources are constrained and disaster response systems are often nascent or insufficiently organized. Traditionally, management has relied on supportive care, stringent infection prevention and control (IPC), surveillance, and rapid containment. In this context, preparedness hinges not only on technical capacity, but also on the speed with which clinical knowledge and field experience are mobilized. Consequently, the declaration of an MVD outbreak in Ethiopia constituted a critical juncture, with profound implications for healthcare workers and the national health system.

The 2024 Rwandan outbreak marked a paradigm shift in disease management, as several investigational therapeutics and interventions were deployed under emergency and compassionate-use frameworks [3,4]. This period was defined by rapid operational learning and real-time knowledge synthesis among frontline clinicians. However, when Ethiopia, a nation of 130 million in East Africa with no prior history of MVD, faced this outbreak, many frontline clinicians encountered significant gaps in clinical awareness and systemic preparedness. This vulnerability was exacerbated by the daunting global context of MVD’s high mortality and a lack of definitive medical countermeasures.

To mitigate this crisis, the Ethiopian Society of Emergency Professionals (ESEP) rapidly organized a series of national webinars linking Ethiopian health professionals with Rwandan frontline responders. Rwanda’s success in containing the virus, achieving a significantly reduced case fatality rate (CFR) of 22.7% (rounded to 23%) compared to historical benchmarks, provided an invaluable pedagogical opportunity. Within 24 hours of the outbreak declaration, these sessions were launched nationwide, reaching hundreds of healthcare professionals and continuing on a weekly basis thereafter.

These webinars bridged the gap between theory and practice, transitioning from foundational IPC documentation to technical “deep-dives” into triage, fluid resuscitation, and electrolyte management—critical skills informed by Rwanda’s recent clinical successes. This knowledge translated to bedside practice, empowering Ethiopian clinicians to implement immediate, life-saving interventions.

Beyond technical guidance, the sessions incorporated survivor testimonials to address the psychosocial well-being of frontline staff. This “hit hard, hit early” clinical philosophy, delivered through sustained bidirectional dialogue, fortified healthcare professionals both clinically and psychologically. This impact was reflected in trainee feedback, which described increased confidence and a heightened state of preparedness to tackle the outbreak. By offering cost-free access to expert insights, these sessions demonstrated how timely, society-led initiatives can significantly bolster response capacity even before international support arrives.

This South–South collaboration functioned as an effective first line of defense, preceding formal international guidance and enabling the exchange of protocols. A key component of this success was the formal endorsement of these documents by local institutions, ensuring that healthcare providers viewed them as locally owned rather than externally imposed. This synergy was bolstered by a long-standing history of friendship, notably Ethiopia’s support in training over 100 Rwandan resident doctors in Addis Ababa to support health workforce development under Rwanda’s 4x4 reform agenda, which created a foundation of trust vital for real-time knowledge exchange.

As Marburg outbreaks continue to emerge in the region, Rwanda’s experience, marked by the lowest case fatality rate of 22.7% (rounded to 23%), and the rapid peer-to-peer collaboration that followed, offers a compelling case for re-centering outbreak preparedness and response around empowered regional networks, frontline leadership, and sustained South–South partnerships. These actors are not merely adjuncts to global response systems; they are, in many cases, the real first responders. Moreover, such collaboration lays the foundation for risk communication strategies and community engagement, creating a platform for sharing experiences and building vital trust among the affected community groups.

The recurrent emergence of MVD in East Africa underscores the urgent need to recalibrate global outbreak preparedness frameworks, particularly in settings that share similar cultural contexts and resource constraints. To be effective, policymakers, multilateral agencies, and donors must formally recognize and invest in South–South collaboration as a core pillar of epidemic response. This includes:

- **Sustained resourcing** of regional medical societies and professional associations;
- **Embedding** frontline clinicians from affected countries into real-time decision-making, guideline development, and research governance;
- **And accelerating** regulatory pathways for the ethical deployment of investigational therapeutics during outbreaks.

Long-term investment in regional research infrastructure, workforce protection, and cross-border training is essential to ensure that future filovirus outbreaks are met with responses that are faster, more equitable, and locally led. This paper demonstrates the profound impact of formalizing South-South collaborations. By establishing professional societies as technical implementing partners, global health actors can effectively decentralize outbreak management through robust regional networks.

Targeted and intentional funding of these local structures offers a clear pathway toward sustainability, particularly given the recurrent nature of public health threats in Africa. Regions navigating similar socio-economic hurdles are uniquely positioned to develop context-specific solutions that do not compromise the standards of global health delivery. Therefore, multilateral organizations such as the World Health Organization should consider formally integrating and funding these initiatives to optimize resource utilization and regional expertise. Such investment is a critical step toward ensuring sustainable vigilance and enabling timely, expert-led responses.

Ultimately, these professional partnerships constitute a scalable, cost-effective, and underutilized mechanism for optimizing knowledge exchange. As regions face increasingly frequent and similar outbreaks, fostering internal disaster preparedness alongside cross-border support is not merely an option: it is an essential driver for advancing global health equity and mitigating preventable mortality [5].

While every approach has limitations and “one size fits all” solutions are rare, the success of such collaborations often hinges on the strength of local networks and the reality of resource constraints. Furthermore, many collaborative frameworks remain under-explored. Yet, the recent joint response between these two “all-weather friends,” Ethiopia and Rwanda, serves as a compelling model for this paradigm, demonstrating the profound impact of regional solidarity when facing a shared viral threat.
